# Route and antigen shape immunity to dmLT-adjuvanted vaccines to a greater extent than biochemical stress or formulation excipients

**DOI:** 10.1016/j.vaccine.2023.01.033

**Published:** 2023-01-31

**Authors:** Addison E. Stone, Saraswatie Rambaran, Ivy V. Trinh, Marcus Estrada, Curtis W. Jarand, Blake S. Williams, Amelie E. Murrell, Chelsea M. Huerter, William Bai, Surya Palani, Yukihiro Nakanishi, Renee M. Laird, Frederic M. Poly, Wayne F. Reed, Jessica A. White, Elizabeth B. Norton

**Affiliations:** 1 Department of Microbiology & Immunology, Tulane University School of Medicine, New Orleans, LA; 2 PATH, Seattle, WA, USA; 3 Department of Physics and Engineering Physics, Tulane University School of Medicine, New Orleans, LA; 4 Department of Pathology. Moffitt Cancer Center, Tampa, FL; 5 Henry M. Jackson Foundation for Military Medicine, Bethesda, Maryland, USA; 6 Enteric Diseases Department, Naval Medical Research Center, Silver Spring, Maryland, USA

**Keywords:** adjuvant, formulation stability, vaccine efficacy, dmLT, mucosal immunity, ETEC, polio

## Abstract

A key aspect to vaccine efficacy is formulation stability. Biochemical evaluations provide information on optimal compositions or thermal stability but are routinely validated by *ex vivo* analysis and not efficacy in animal models. Here we assessed formulations identified to improve or reduce stability of the mucosal adjuvant dmLT being investigated in polio and enterotoxigenic *E. coli* (ETEC) clinical vaccines. We observed biochemical changes to dmLT protein with formulation or thermal stress, including aggregation or subunit dissociation or alternatively resistance against these changes with specific buffer compositions. However, upon injection or mucosal vaccination with ETEC fimbriae adhesin proteins or inactivated polio virus, experimental findings indicated immunization route and co-administered antigen impacted vaccine immunogenicity more so than dmLT formulation stability (or instability). These results indicate the importance of both biochemical and vaccine-derived immunity assessment in formulation optimization. In addition, these studies have implications for use of dmLT in clinical settings and for delivery in resource poor settings.

## INTRODUCTION

Vaccine success is a combination of multiple factors including efficacy, manufacturing ease, stability, delivery [1]. Adjuvants are a common method to enhance immunogenicity of vaccines. Certain adjuvants, like aluminum hydroxide, can even enhance stability of vaccine formulations [2], while others can help promote immunity with systemic or mucosal delivery like the double mutant of heat-labile enterotoxin (LT) from enterotoxigenic *E. coli* (ETEC) LT-R192G/L211A or dmLT [3, 4].

dmLT is an 84-kD protein composed structurally in a AB_5_ conformation [3, 4]. Unlike pattern recognition receptor ligand adjuvants, dmLT activates innate immunity including MHC-II and cytokine secretion through combined activities of its A- and B-subunits [5–7], The A-subunit (28kDa) is enzymatically active and associated noncovalently with its pentameric B subunit, composed of five 11.5kDa monomers. The enzymatic A-subunit will ADP-ribosylate host protein Gsα, which causes adenylate cyclase activation and a subsequent increase in intracellular cAMP levels [8–12]. There are two amino acid residue substitutions in the A-subunit that differentiate dmLT from its parent molecule. These alter its enzymatic and secretory properties in epithelial cells but maintain dmLT/LT immune stimulating properties in antigen presenting cells and adjuvant activity [3, 13–17].

dmLT is a toxoid and can function as an antigen to confer protection against LT-mediated diarrheal disease from LT+ ETEC strains [12, 18, 19]. However, it has mainly been utilized for its adjuvant properties: dmLT improves mucosal and parental immunity to viral and bacterial antigens in various routes in animal models, including intramuscular (IM), intradermal (ID), and sublingual (SL) delivery routes [6, 13, 20–24]. dmLT by itself has been proven safe and effective in human clinical trials by oral or sublingual delivery [25, 26] and intradermal or intramuscular delivery [27, 28]. In Phase 1 and 2 studies with oral live-attenuated or inactivated whole cell ETEC vaccines in adults and infants, dmLT promoted mucosal IgA responses to both protein and polysaccharide antigens in these vaccines [29], reduced challenge strain shedding, and was associated with protection from challenge [14, 16, 30]. Recently, dmLT has also been tested with a subunit ETEC vaccine using fimbrial tip adhesin (FTA) protein antigens by injected delivery in animals [24, 31–34] and humans [27]. This includes CfaEB and CssBA which respectively target CFA/I and CS6 colonization factor expressing ETEC bacterial strains. dmLT is also being explored to enhance dose sparing and mucosal immunity as an injected adjuvant in combination with the existing inactivated polio vaccine with promising results in mice models [35] and humans [28].

Vaccine stability and ease of delivery are critical components for a vaccine, especially in low resource settings. Limited thermostability of a vaccine can constrain wide-spread use and increase the cost of the vaccine by requiring cold transport and storage [36]. As dmLT is being investigated for use in multiple vaccine candidates targeting low- and middle-income countries for adults as well as children under 5 years of age ([3, 4, 14, 27, 28, 30, 37]), identifying formulations which improve vaccine and adjuvant thermostability will be critical for future vaccine success. Current dmLT pre-clinical and clinical use involves resuspension of a lyophilized product and bedside missing with antigen and aqueous buffer prior to immediate vaccination. However, final vaccine production will likely require a stable, liquid formulation.

Vaccine production requires standardization with minimal variation in the product’s chemical and physical properties. Recent dmLT biochemical studies have evaluated optimal conditions for maintaining the bulk protein liquid stability to minimize any protein aggregation or subunit dissociation by testing inclusion of sugars, surfactants, and pH changes to optimize thermal and physicochemical stability of dmLT [38, 39]. These studies confirmed that neutral pH, phosphate, sodium chloride, sugars and/or amino acid buffer components/excipients provide optimal biochemical stability. However, none of these studies have examined the effect of these formulations changes on vaccination including dmLT adjuvant activity or immunogenicity (relevant specifically for ETEC vaccines). We have previously noted that lactose-containing formulations of dmLT injected intradermally modified local skin reactogenicity but not development of cellular and humoral responses in a mouse model of ETEC FTA subunit vaccine [24]. This would suggest that formulation changes may have unanticipated and even beneficial effects on desired vaccine outcomes that should be better understood. Here, we perform a rigorous evaluation to investigate how biochemical stressors and formulation excipients alter dmLT biochemistry and induction of humoral immunity using pre-clinical models of polio and ETEC vaccines as well as parenteral or mucosal delivery routes.

## MATERIALS & METHODS

### Vaccine Antigens.

Inactivated polio vaccine, IPOL 80DU/ml (Sanofi Pasteur) was purchased from Tulane Pharmacy. IPOL was diluted with dmLT formulations in Media-199 (Gibco). dmLT was produced from *E. coli* clones expressing recombinant protein derived from the human ETEC isolate *E. coli* H10407 as previously described in our laboratory [4]; or dmLT was produced according to GMP (Good Manufacturing Practices) specification by IDT in sodium phosphate buffer supplemented with 5% lactose as a lyophilized product in vials containing 700 μg product in a 3 ml sterile, multidose, Wheaton serum vial stored at −20°C. CfaEB and CssBA were expressed and purified from a BL21(DE3) expression host using previously described methods [40, 41]. Briefly, CssBA was purified from the clarified cell lysates using a Ni-NTA superflow column, and the protein was eluted with a step imidazole gradient (135 mM imidazole over 10 column volumes) in a 20 mM phosphate buffer pH 7.4, followed by a HiTrap SP HP column, in which the protein was eluted with a step salt gradient (140 mM sodium chloride over 5 column volumes) in a 20 mM phosphate buffer, pH 6.3. CfaEB was purified from the clarified cell lysates using a Ni-NTA superflow column, and the protein was eluted with a step imidazole gradient (100 mM imidazole over 8 column volumes) in a 20 mM Tris buffer pH 8.0, followed by a HiTrap SP HP column, in which the protein was eluted with a step salt gradient (250 mM sodium chloride over 8 column volumes) in a 20 mM succinate buffer, pH 5.0. Protein antigens were stored frozen until use.

### dmLT formulations.

dmLT was resuspended from lyophilized protein to a concentration of 1mg/ml. Buffers with specific pH, sugar content (lactose, sucrose), amino acids, and/or polysorbate-80 surfactant were made fresh according to the formulations listed in Table 1. For polio vaccine studies, dmLT was diluted into formulation buffers resulting in a 50-fold dilution of the lyophilization excipients. For ETEC vaccine studies and ARGEN experiments, dmLT was dialyzed in respective buffers using a Slide-A-Lyzer mini-Dialysis Device (ThermoFisher) for 3 cycles of dialysis at 4C with fresh buffer. Next, dmLT was collected and quantified using a spectrophotometer or BCA assay (Pierce) then diluted to 0.15 mg/ml. Various formulations were stored for 1–7 days at 2–8°C or 40°C as specified. Afterwards, dmLT was removed from storage/thermal stress for formulation or immunization analysis. Vaccine formulations for polio mouse immunizations were frozen for transport first whereas formulations for ETEC studies were used immediately. After immunizations all solutions were diluted 1:1 in 200 μl PBS-PS80 and shipped to PATH for dmLT dose verification in the GM1 binding ELISA.

### Mouse Immunizations.

Female BALB/c mice, 6–8 weeks of age, were purchased from Jackson Laboratories (Strain #:000651) and housed in sterile cages. All animal studies were approved by the Tulane University Institutional Animal Care and Use Committee. For ID and IM immunizations, formulations were injected with a 0.3 cc needle into the shaved, hind flank skin (ID) by Manteaux technique or directly into the caudal thigh muscle (IM), alternating right or left sides with each immunization. For SL immunization, mice were intraperitoneally injected with ketamine/xylazine, held upright for 1mn while 18 μl formulation was pipetted underneath the tongue, rested for 2–4 minutes, and then repeated with another 18 μl for a total of 36 μl delivered SL. Immunizations were performed 2–3 times at a 3-week interval. Fecal pellets were freshly collected from live mice at week 5 a day prior to CO_2_ euthanasia and exsanguination for blood collection. Blood was centrifuged for serum and subsequently stored at −20°C until ELISA analysis. Fecal pellets were weighed, homogenized in 1.5 ml PBS − 0.05% Tween 20 containing a protease inhibitor cocktail (Roche Diagnostics, Indianapolis, IN), and the supernatants collected at stored at −20°C until ELISA analysis.

### Skin Analysis.

Immunization site skin was excised carefully to avoid any tissue disruption and measured for gross thickness (mm) by digital calipers and then fixed in 10% formalin, followed by paraffin embedding, sectioning, and staining by H&E. Skin samples were blindly scored by a board-certified pathologist for type (1=acute/neutrophilic infiltration, 2=chronic/lymphoplasmacystic infiltration, 3=both), severity (1=minimal/mild, 2=moderate, 3=severe), location (1=dermis, 2=subcutis, 3=both), and distribution of inflammation (1=localized, 2=diffuse). Composite of these scores were used as the final histology score.

### SDS-PAGE.

Samples were loaded into wells of a 4–12% Bis-Tris NuPAGE gel (ThermoFisher Sci.) for gel electrophoresis, stained with Coomasie Blue, and imaged for picture or band density using Amersham Imager 600. Percentage of A, B_1_, B_5_ band densities were calculated per lane. dmLT formulations were assayed from frozen samples in PBS-0.1% polysorbate 80 buffer (polio experiments) or fresh samples (ETEC experiments) that also matched formulations used for immunizations.

### GM1 binding ELISA.

ELISAs were performed as previously described [42]. Briefly a monoclonal antibody against the A-subunit of dmLT was used for detection of intact dmLT molecules bound to GM1. Detection antibody (Goat Anti-Mouse IgG γ chain) was prepared by diluting 1:8,000 in Dulbecco’s phosphate buffered saline (DPBS Fisher Scientific) with 1% bovine serum albumin (w/v) (Thermo Fisher Scientific) and 0.05% Tween 20 (w/v) (Fisher Scientific). All ELISAs were performed on dmLT formulations used for mouse immunizations that were frozen for transport in PBS-0.1% polysorbate 80 buffer.

### ARGEN Light Scattering.

The stability of dmLT under thermal stress in various buffers sytems has been studied by ARGEN (Fluence Analytics, Houston TX) at the Center for Polymer Reaction Monitoring and Characterization (PolyRMC) at Tulane University. ARGEN is a static light scattering instrument that consists of 16 sample cells with independent thermal and stirring controls. Samples of dmLT were analyzed as received, following filtration through a 0.22 μm cellulose acetate syringe filter with a final volume of 1ml following published methodology [43]. Briefly, the Aggregation Rate (AR) to measure thermal stability was determined from the early linear regime of the aggregation profile, *Time to Equivalent Dimerization (TED)* or (I/Kc) were readouts from this analysis. AR is calculated using the dimensionless quantity ${\text{M}}_{\text{w}}\left(\text{t}\right)/{\text{M}}_{0}$, where ${\text{M}}_{\text{w}}\left(\text{t}\right)$ is the weight average molar mass of all scatterers in solution at time t after a stressor is applied. ${\text{M}}_{\text{w}}\left(\text{t}\right)$ includes all native proteins and aggregates, and ${\text{M}}_{0}$ is the molar mass of the native, unaggregated protein. ${\text{M}}_{\text{w}}\left(\text{t}\right)/{\text{M}}_{0}$ hence represents the equivalent number of proteins per aggregate. The aggregation rate (AR) defined as $AR\left(s^{-1}\right)={\left.\frac{d\left[M_{w}\left(t\right)/M_{0}\right]}{dt}\right|}_{t=0}$. To relate AR to parameter relevant to vaccine stability, the TED at a certain temperature (T), such as T=40 °C, or any other temperature, was calculated. The TED is the amount of time it takes for initially non-aggregated protein to aggregate to a level such that the average molecular weight of the non-aggregated and aggregated populations together is equal to the molecular weight of a dimer of the protein. It was also possible to measure an effective weight average molecular weight M_w,eff_=I/Kc. In this, c is the concentration (g/ml) of the protein in solution, K is an optical constant which uses dn/dc=0.189 cm^3^/g for the protein in aqueous solution and also contains the 660nm incident laser wavelength. I is the Rayleigh Scattering ratio, obtained by referencing the scattering intensity of the protein solutions to the known Ralyeigh Ratio of pure toluene at 25°C and 660nm; I_toluene_(660 nm)=1.18×10^−5^ cm^−1^. Because the native form of dmLT is low mass with a radius of gyration less than 10nm, there is no significant angular dependence to the scattering, so that ARGEN measurements at 90 degree scattering angle is sufficient for M_w,eff_ determination. Similarly, the low concentration of the dmLT leads to very little interprotein interaction, so that excluded volume effects, embodied in the second virial coefficient, are negligible. All dmLT formulations were assayed from fresh samples, for ETEC experiments this also matched formulations used for immunizations.

### Antibody enzyme-linked immunoassay (ELISAs).

Fecal and sera IgG and IgA antibody ELISAs were performed using Corning 96 well flat bottom plates (Costar 9018). They were coated with 0.1μg/well dmLT, CS6, CFA/I, or 1.83DU/well IPOL (Sanofi Pasteur) and were quantified with dilutions of purified mouse recombinant standards IgG1 (Millipore Sigma) or IgA (Southern Biotech, Birmingham, AL). CS6 and CFA/I were obtained through BEI Resources, NIAID, NIH, respectively BEI NR-49116 and BEI NR-49109. ELISAs were detected using AKP-conjugated anti-mouse IgG or HRP-conjugated anti-mouse IgA (Sigma). Plates were developed for IgG and IgA after serum and fecal incubation. Results were expressed as ELISA units/ml (EU/ml) using averaged interpolated values around the standard curve midpoint and log2 transformed.

### Polio Neutralization Assay.

Samples were tested using a standard microneutralization assay for antibodies to oral poliovirus types 1, 2, and 3 Sabin strains (kindly provided from Dr. Steven Oberste, Polio and Picornavirus Laboratory Branch Chief, CDC). Protocols were adopted from established protocols at the Global Polio Specialized Laboratory, Centers for Disease Control and Prevention [44]. Briefly, 80–100 CCID50 of each poliovirus serotype and 2-fold serial dilutions of serum were combined and pre-incubated at 35°C, 5% CO_2_ for 3 hours before addition of HEp-2(C) cells (50,000 cells per well) in 96-well tissue culture treated flat bottom plates (CytoOne). After incubation for 5 days at 35°C and 5% CO_2_, plates were stained with Neutral Red (Sigma) and imaged by Immunospot ELISPOT Analyzer. Each specimen was run in duplicates, with parallel specimens from one study subject tested in the same assay run, and the neutralization titers estimated by the Spearman-Kärber method and reported as the reciprocal of the calculated 50% end point titer (e.g. serum dilution factor) and log2 transformed. Reference antiserum pool was used to monitor assay performance and variation. A serum sample was considered positive if antibodies were present at ≥1:8 dilution. Specimens with antibody titers <1:8 were considered seronegative and given a log2 value of 2.

### dmLT structure.

The structure of dmLT were derived from the previously published structure of partially-activated *E. coli* heat-labile enterotoxin, PDM ID: 1LTB [45], using Jmol: an open-source Java viewer for chemical structures in 3D (http://www.jmol.org/).

### Statistics.

Statistical analyses were performed using Prism (GraphPad Software v9). Values are represented as mean + SEM with significance indicated as *P ≤ 0.05, **P ≤ 0.01 or ***P ≤ 0.001. Parametric data was analyzed by one-way ANOVA using Bonferroni’s multiple comparison or Kruskal-Wallis uncorrected Dunn’s post-hoc test. Data were tested to confirm lack of normality (D’Agostino & Pearson) and then tested by Spearman correlation. Percentage of dmLT (no stress) control group was calculated using the average antibody or neutralization data of the dmLT no stress group after first subtracting the baseline calculated from the average of the naïve group and then tested by one-way ANOVA using Bonferroni’s multiple comparison or Kruskal-Wallis uncorrected Dunn’s post-hoc test.

## RESULTS

### Injection volumes have modest effects on dmLT vaccination.

**Figure 1.**
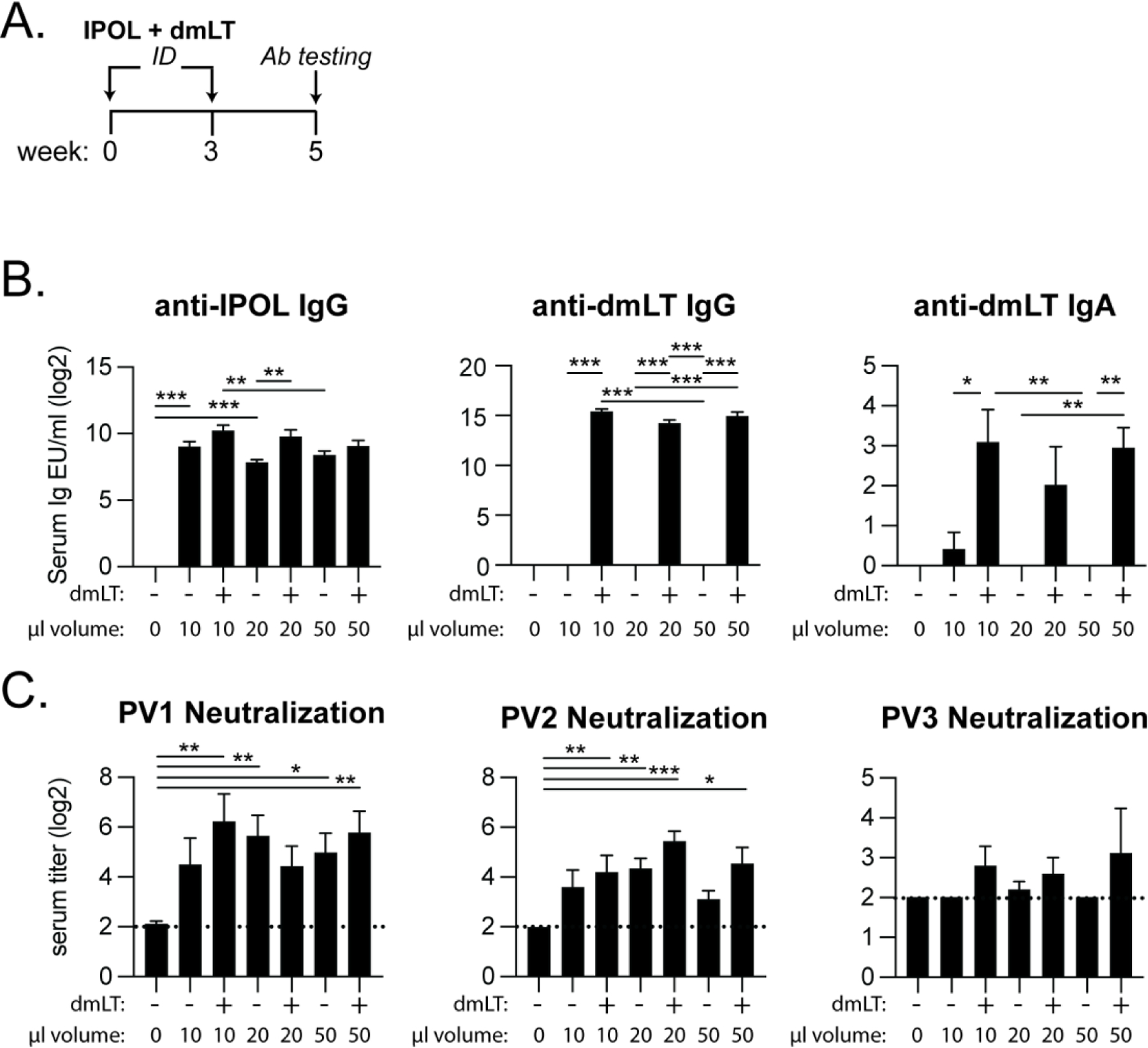
Poliovirus serum IgG, IgA, and neutralization titers following intradermal administration of 0.1 μg dmLT in various immunization volumes. BALB/c mice (n = 5) were either left naïve or immunized twice (week 0 and 3) with 1 DU of IPOL by ID delivery with or without 0.1 μg dmLT in 10 μl, 20 μl, or 50 μl of M-199. Serum was collected 2 weeks after the last immunization and analyzed by ELISA or *in vitro* virus neutralization. **(A)** Schematic of immunization schedule and antibody (Ab) testing. **(B)** Serum anti-IPOL IgG, anti-dmLT IgG, and anti-dmLT IgA graphs for log2 transformed ELISA units (EU)/ml sample. **(C)** *In vitro* virus neutralization titers against each vaccine poliovirus strains PV1, PV2, or PV3 as the inverse of serum dilution factor log2 transformed. Dotted line placed at the naïve mouse response (background). Values are represented as mean + SEM with significance indicated as *P ≤ 0.05, **P ≤ 0.01 or ***P ≤ 0.001 by ANOVA using Bonferroni’s multiple comparison (ELISA) or Kruskal-Wallis uncorrected Dunn’s (neutralization) post-hoc test.

Prior to testing the effects of dmLT formulations with biochemical stress, we wanted to optimize intradermal delivery volume as these have differed in past reports [20, 24, 35]. To do so, we administered dmLT intradermally in conjunction with IPOL, a commercial human polio virus vaccine similar to past studies [35]. We immunized mice with 10, 20, or 50μl volumes with or without 0.1μg of dmLT adjuvant and 1DU IPOL on week 0 and 3 (Fig. 1A). Two weeks after the last immunization, we evaluated anti-IPOL and anti-dmLT serum IgG and IgA antibodies by ELISA. Antibody levels were similar between administration volumes. We found a significant improvement in the development of antibodies when the intradermal dose included 0.1μg of dmLT in a 20μl dose (Fig. 1B). When analyzing neutralization titers via neutralization assay to 3 Sabin serotypes polio virus strains (PV1, PV2, and PV3), we found no statistically significant impact of dmLT dosage (Fig. 1C). Based on the results of this pilot experiment, we chose the delivery volume to be dmLT at 0.1 μg in 20μl for the rest of our IPOL experiments.

### Biochemical stability of dmLT is altered by formulation excipient sugars, surfactants, pH and thermal stress.

**Figure 2.**
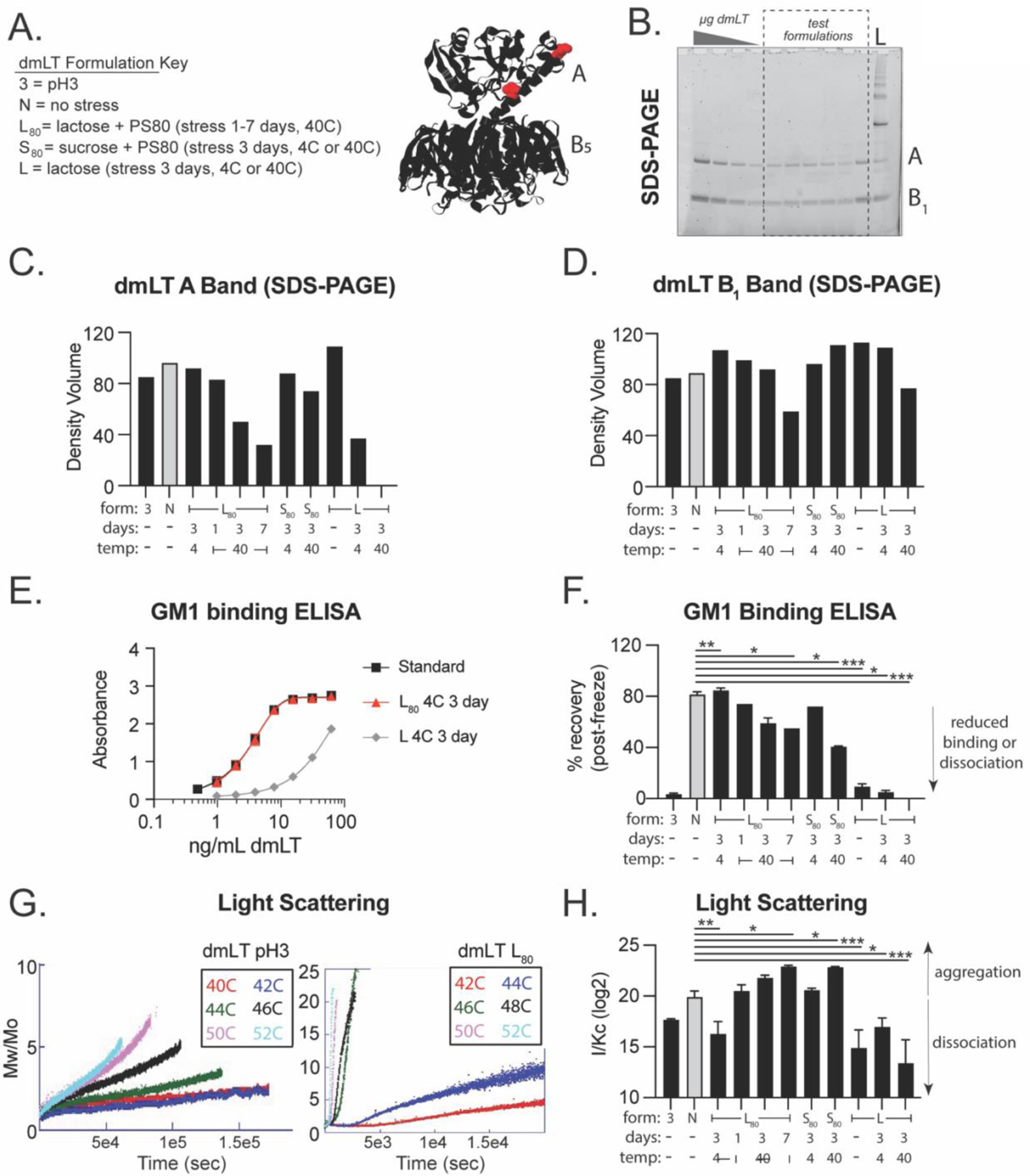
dmLT formulation optimization via biochemical analyses post thermal or chemical stressor to be used in Polio model. **(A)** Formulation key and dmLT amino acid backbone visualization with R192G and L211A mutations highlighted in red. **(B)** Representative images of SDS-PAGE analysis of dmLT, polio dmLT formulations or ladder (L) with A and B bands indicated. **(C)** A band density quantification from SDS-PAGE analysis. **(D)** B_1_ band density quantification from SDS-PAGE analysis. **(E)** Representative GM1 binding ELISA. **(F)** GM1 Binding ELISA quantified as % dmLT recovered from standard, tested in triplicate using polio vaccination formulations. **(G)** ARGEN light scattering evaluation of dmLT average molar mass of changes over time indicating aggregation behavior of dmLT in PBS at pH 3.1 (left) and L_80_ (right) buffers (made freshly, independent of vaccination experiments). **(H)** Light Scattering quantification indicating aggregation or dissociation of dmLT molecules for representative formulations tested in triplicate. For all bar graphs, grey bars indicate no stress dmLT group and values are represented as mean + SEM with significance indicated as *P ≤ 0.05, **P ≤ 0.01 or ***P ≤ 0.001 by ANOVA using Bonferroni’s multiple comparison (ELISA) or Kruskal-Wallis uncorrected Dunn’s (light scattering) post-hoc test.

Previous reports have identified dmLT formulations that create optimal thermal and physicochemical stability [38, 39] for maintaining its AB_5_ configuration (Fig. 2A) but have not evaluated this concurrently in vaccination models. To test how biochemical stressors can impact stability of dmLT, we evaluated dmLT in buffers containing variations of low pH (abbreviated as 3), polysorbate-80 surfactant (PS80), and either lactose (L) or sucrose (S). These excipients were identified to alter dmLT aggregation, chemical degradation, particle formation, and conformational destabilization [38]. Lactose containing formulations were also used in the first GMP production of dmLT [25]. Based on this, we anticipated that a low pH and thermal stress applied to formulations without PS80 would be less optimal; however, L or S sugar containing formulations with PS80 would enhance dmLT biochemical stability and preserve adjuvant activity. dmLT was added to formulations containing the typical PBS buffer, or modified as indicated in Table 1 for pH3, L, L+PS80, S+PS80 and stored for 0–7 days at 4 or 40°C prior to analysis. dmLT stability in the selected formulations were evaluated by SDS-PAGE, GM1 binding ELISA, or ARGEN light scattering. SDS-PAGE gel analysis after boiling showed presence of an A band (28kD) and a B_1_ monomer band (11.5kD) band as expected [4]. Thermal stress induced noticeable degradation of these A and B bands (40°C over 3–7 days) compared to the no stress group (N, grey bars; Fig. 2B–D). GM1 binding ELISA detects dmLT protein in an AB5 configuration by first capturing the B-subunit to GM1-coated wells and next using antibody detection of the A-subunit [42]. Results of this GM1 binding ELISA indicate a similar trend as SDS-PAGE, in that thermal stress reduced recovery of AB_5_ dmLT protein but also clearly show pH3 buffer results in almost complete loss of dmLT’s AB_5_ structure (Fig.2E–F). Lastly, ARGEN light scattering was used to quantify aggregation or dissociation [46, 47]. In this analysis, S+PS80 or L+PS80 and thermal stress significantly induced aggregation, whereas pH3 and L (without PS80) resulted in significant disassociation of the AB_5_ molecule using the I/Kc relative measure of molecular weight (Mw; Fig.2G–H) and aggregation rate or time to equivalent dimerization analysis (Suppl. Fig.1). However, even dmLT in a pH3 buffer retains some ability to stimulate immune cells, as the human monocyte cell line THP-1 cells will undergo partial activation with MHC-II expression after treatment, though not to the extent of non-stressed dmLT (Suppl. Fig.1).

As anticipated, these results show that dmLT in a pH3 buffer results in dissociation of the A and B subunits. L or S sugars + PS80 show better thermal stability of the AB_5_ configuration than sugar only formulations, but the newer analysis by light scattering identifies there is also more aggregation of the protein during this process.

### Addition of a PS80 surfactant has minimal changes on skin after intradermal injection with dmLT adjuvanted polio vaccine.

**Figure 3.**
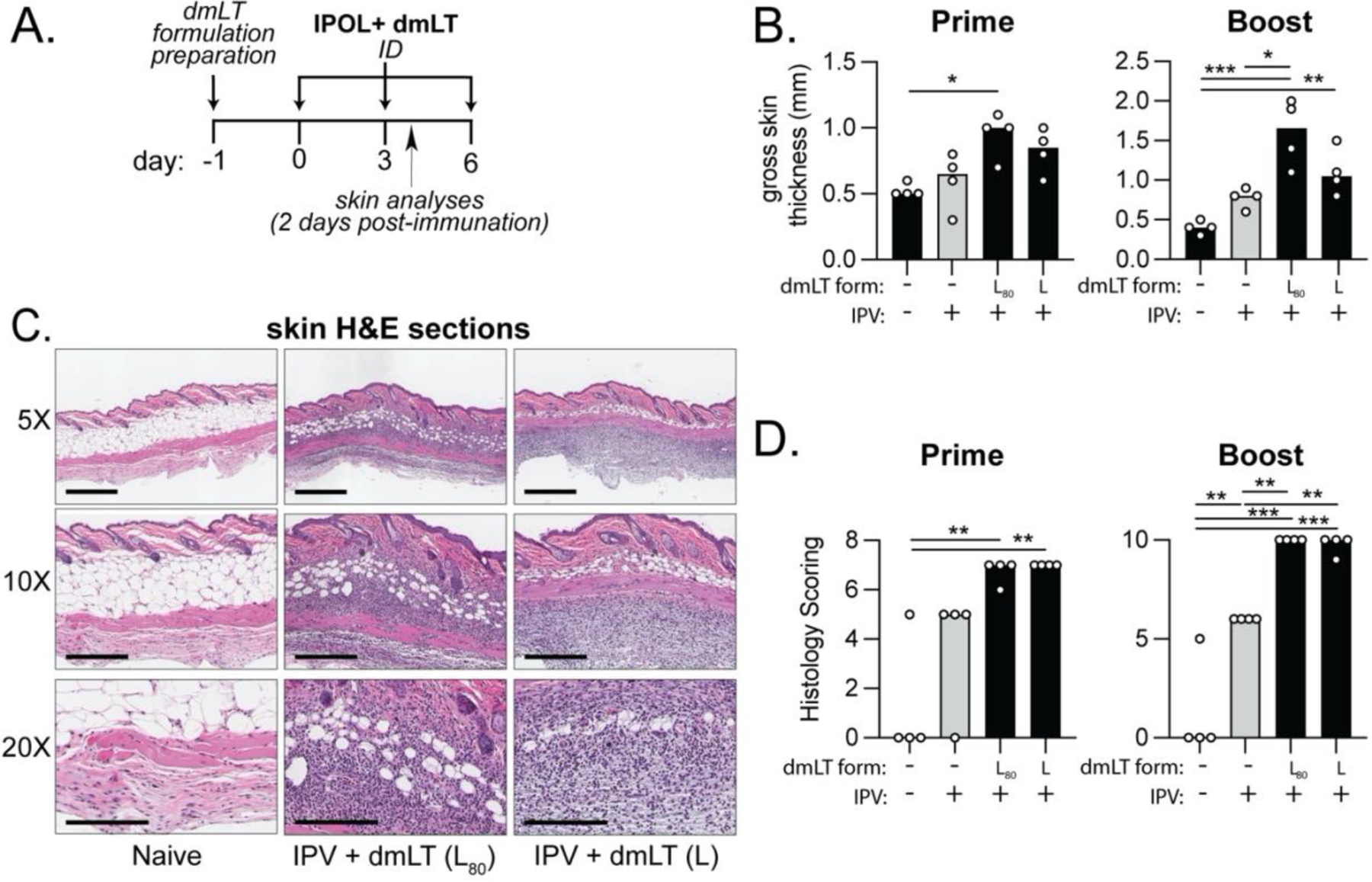
Skin reactogenicity post intradermal immunization with dmLT formulations having undergone thermal and chemical modifications in the Polio vaccination model. dmLT formulations were combined with for 3 days before vaccination and held at 4C. BALB/c mice (n= 4–5) were then immunized with 1DU of IPOL alone or in conjunction with 0.1μg of dmLT at week 0, boost on week 3, and euthanized 2 days post-immunization to analyze skin reactogenicity post immunization. dmLT formulations included L_80_ (lactose + PS-80, 4C for 3 days) and L group (lactose, 4C). **(A)** Schematic of timeline for formulation preparation and animal immunizations. **(B)** Skin reactivity at intradermal injection site quantified by gross skin thickness in millimeters. **(C)** Representative H&E-stained images of the skin at the injection site of mice treated with dmLT formulations as indicated. Scale bars represent 500μm, 300μm, and 200μm for 5X, 10X, and 20X respectively. **(D)** Skin reactivity at intradermal injection site quantified by gross skin thickness in millimeters. Values are represented as mean + individual points with significance indicated as *P ≤ 0.05, **P ≤ 0.01 or ***P ≤ 0.001 by ANOVA using Bonferroni’s multiple comparison post-hoc test.

It has been shown that biochemical stress (e.g., heat, pH) to dmLT formulations can impact stability (Fig.2) [38, 39], the effects of these stressors have not been validated in an animal model of vaccination. We previously noticed that lactose improved skin reactogenicity in intradermal vaccination with dmLT [24]; however, this has not been assessed for PS80 which as a surfactant could disrupt tissue integrity. To investigate this further, we prepared formulations as shown in Table 1 and kept some formulations at either 4 or 40C for 0–7 days. Afterwards, dmLT formulations were used to immunized mice with co-administered IPOL on week 0 or 3. Specific groups of mice were analyzed 2 days after the second immunization for skin changes at the prime site or booster site by gross measurement or blinded histological scoring of H&E sections as shown (n=4 per group, Fig. 3C, Suppl. Fig.2). Skin thickness was significantly increased in both prime and booster skin sections in the mice immunized with IPOL combined with dmLT formulated with L+PS80 (pre-stored for 3 days at 4C; Fig. 3B) compared with naïve mice, IPOL alone or IPOL combined with dmLT formulated with L only (no pre-storage). All IPOL containing immunizations had evidence of histological changes at prime and booster skin tissue sections (Fig. 3C–D); however, this was significant for L and L+PS80 formulations at the booster site, which indicate only acute skin changes 2 days post-vaccination was observed. Thus, all observed skin changes were deemed minor with no evidence of skin ulceration, laceration, or even persistent histological changes after vaccination. This indicates no significant adverse effects were observed with the addition of PS80 to the formulation.

### Thermal stress and pH stress have minimal impact on ability of dmLT to adjuvant intradermal polio vaccine in mice.

**Figure 4.**
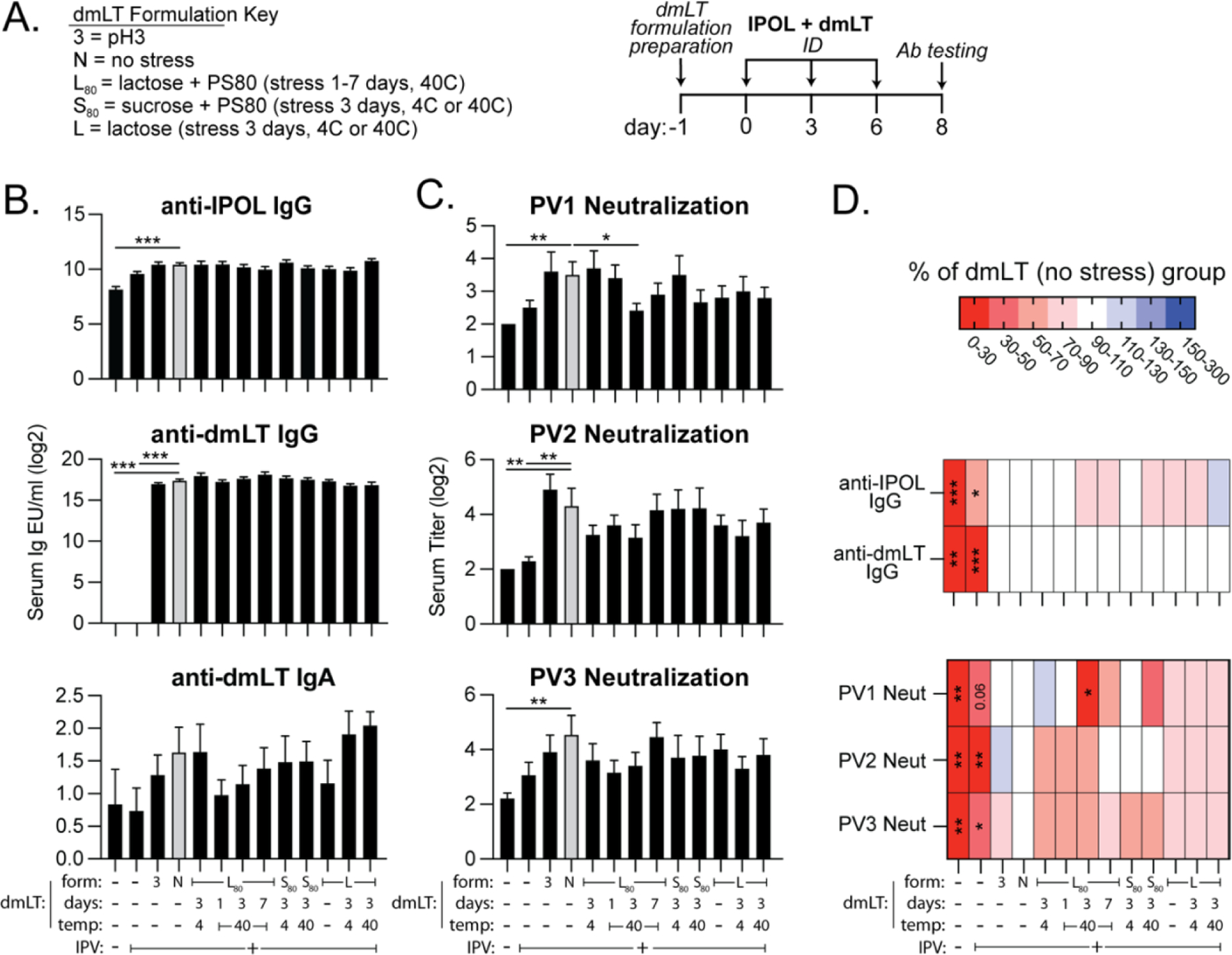
Quantification of immune response in mice immunized with modified dmLT formulations. Various stability excipients were added to dmLT formulations 0–7 days before vaccination and held at either 4C or 40C. BALB/c mice (n= 4–10) were then immunized with 1DU of IPOL alone or in conjunction with 0.1μg of dmLT at week 0, 3 and 6 prior to serum antibody analysis two weeks later (week 8). **(A)** Schematic of immunization schedule and formulation key with indicated dmLT formulation stressors. **(B)** Compiled EU/ml of indicated anti-IPOL Serum IgG, anti-dmLT Serum IgG, and anti-dmLT Serum IgA antibody titers assessed by ELISA and graphed on a log2 scale. **(C)** Compiled serum neutralization to PV type-1 (PV1), PV2, and PV3 polio strains graphed on a log2 scale. (**D)** Percentage change from dmLT no stress group for indicating formulations for ELISA and neutralization data. Bar values are represented as mean + SEM with significance indicated as *P ≤ 0.05, **P ≤ 0.01 or ***P ≤ 0.001 by ANOVA using Bonferroni’s multiple comparison (ELISA) or Kruskal-Wallis uncorrected Dunn’s (neutralization, heatmaps) post-hoc test.

Concurrent to the biochemical and skin analyses (Fig. 2–3), we further tested 11 various dmLT formulations admixed with IPOL for mouse immunization on weeks 0, 3, 6 (Fig. 4A). Two-weeks later mice were euthanized for evaluation of vaccine immunogenicity by serum antibody ELISAs or polio neutralization analyses, the standard analysis for polio vaccination. We quantified serum IgG and IgA responses to IPOL and dmLT by ELISA. No consistent expression of anti-IPOL serum or fecal IgA was detected in any group by ELISA (data not shown). dmLT adjuvanted formulation with IPOL (no stress, N group, grey bars) resulted in significant increase in IPOL and dmLT IgG (Fig. 4B) and polio virus (PV) neutralizing antibody titers to type 1–3 strains (Fig. 4C) compared with naïve mice. We did not find major differences in humoral immunity between dmLT formulations, even with pH3 or formulations pre-stored at 40C for 7 days; although there were some subtle changes in individual neutralizing titers. To show these differences with greater clarity, the % change from the dmLT no stress control group (grey bars) was calculated and shown by heat map. This also indicated that minimal changes occurred in development of IPOL serum IgG, dmLT serum IgG or PV type 1–3 strains neutralization was altered by formulation preparation and stress (Fig. 4D).

**Figure 5.**
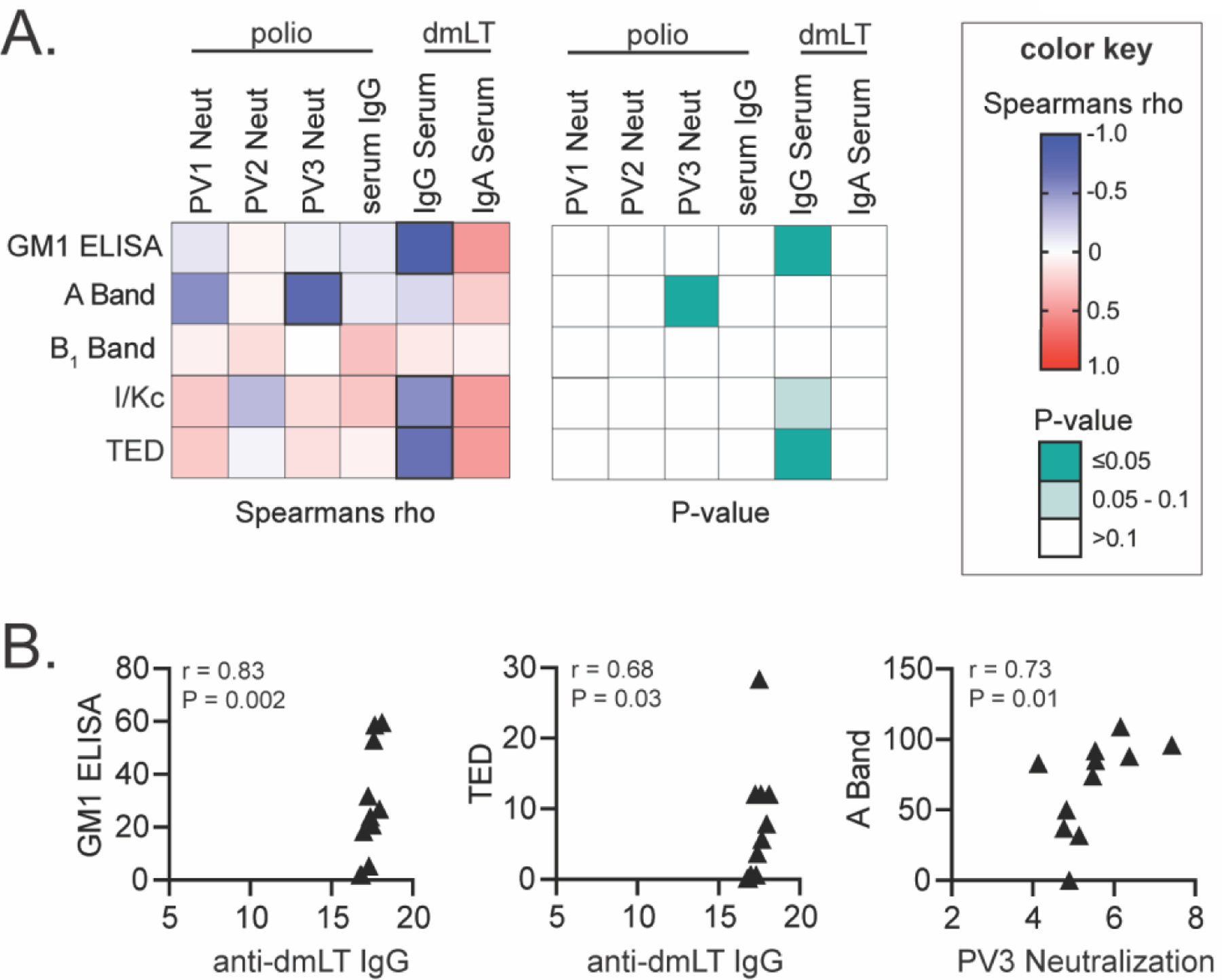
Correlations between formulation modification and immune response in animal model. (A) Heatmaps of Spearman’s rank correlation (rho) and p-values for indicated humoral analysis post-IPOL vaccination and dmLT biochemical analyses. (B) Select graphs of individual correlations with each immunization/formulation group represented by triangle symbol. TED represents the Time to Dimerization of dmLT after analyses on ARGEN Light Scattering machine, and AR shows the correlations of the Aggregation Rate of dmLT to the immune response.

Thus, formulations altering biochemical stability of dmLT (Fig. 2), result in few changes to vaccine efficacy in this polio vaccination model. In addition, subtle responses between polio virus type 1, 2, and 3 neutralization titers differed, as shown when both blue and red bars were present (e.g. pH3, L+PS80 4C). We next correlated formulation stability data with murine antibody response post-vaccination to investigate drivers of vaccine efficacy in this polio model (Fig. 5A–B). Significant, positive correlations were obtained between GM1 binding ELISA and ARGEN light scattering (I/Kc, TED) with development of anti-dmLT serum IgG but not other measures. Polio virus type 3 neutralizing titers were also positively correlated to dmLT SDS-PAGE A band detection. This data further shows that biochemical changes to dmLT by formulation or thermal stress have minimal effects on vaccine adjuvant outcomes, except for immune responses to dmLT itself. Conformational changes to dmLT are likely the major factor for this observation, particularly as antibodies recognize conformational epitopes which has also been observed for enterotoxins [12, 48]. The significant correlation with dmLT A band and PV type-3 neutralization may also suggest that vaccine outcomes may be driven by the antigen (and its combination with dmLT), rather than just dmLT adjuvant stability.

### Administration route is more important than formulation excipient sugars, surfactants, pH changes, or thermal stress in altering the activity of dmLT.

Our studies above suggest biochemical analyses for dmLT are poor surrogate tests for *in vivo* vaccine outcomes. However, we tested only one animal model with the polio vaccine and intradermal delivery. We know that the A-subunit of dmLT can also stimulate antigen presenting cells [7] and is also a potent adjuvant [6, 13, 24, 49, 50]. However, this A-subunit does not have as potent action after oral or sublingual delivery as dmLT [12, 24], indicating route may be a large influence on dmLT activity after thermal or biochemical stress. Thus, to overcome limitation of one animal vaccine model, we performed a second evaluation using FTA subunit ETEC vaccination model with injected and mucosal delivery being used in animal models and clinical trials targeting CS6+ ETEC (CssBA antigen) [27] and CFA/I+ ETEC (CfaE antigen; clinicaltrial.gov Identifiers: NCT01644565, NCT01922856).

**Figure 6.**
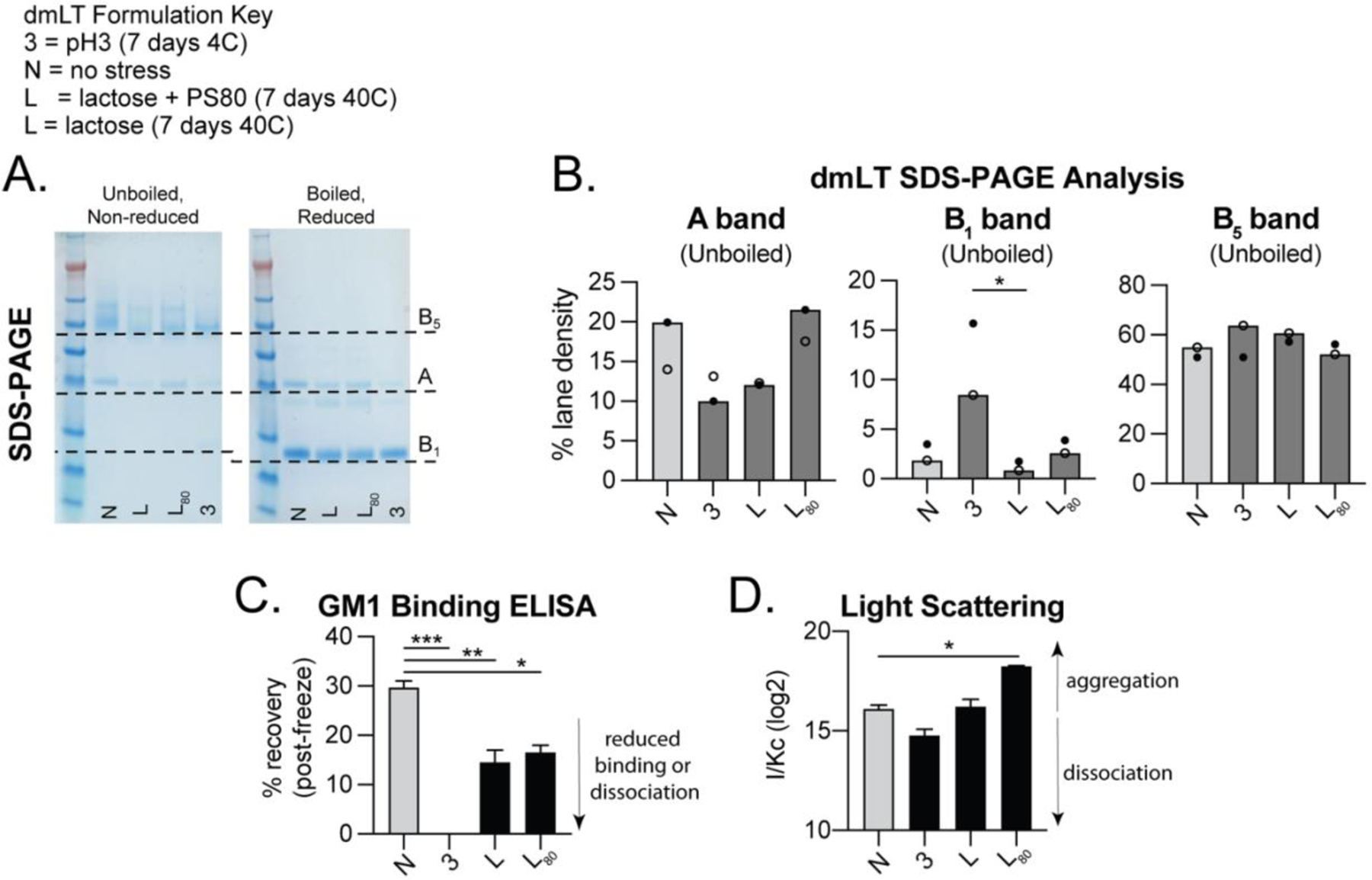
dmLT formulation optimization via biochemical analyses post thermal or chemical stressor to be used in ETEC model. **(A)** Representative image of SDS-PAGE with ETEC formulations loaded and run in unboiled, non-reduced or boiled, reduced conditions, with A, B_1_, and B_5_ bands indicated for dmLT subunits. **(B)** A, B_1_, and B_5_ band density quantification from SDS-PAGE analysis. **(C)** GM1 Binding ELISA quantified as % dmLT recovered from standard, tested in triplicate. **(D)** Light Scattering quantification indicating aggregation or dissociation of dmLT tested in triplicate. Values are represented as mean + SEM with significance indicated as *P ≤ 0.05, **P ≤ 0.01 or ***P ≤ 0.001 by ANOVA using Bonferroni’s multiple comparison (ELISA) or Kruskal-Wallis uncorrected Dunn’s (density, light scattering) post-hoc test.

**Figure 7.**
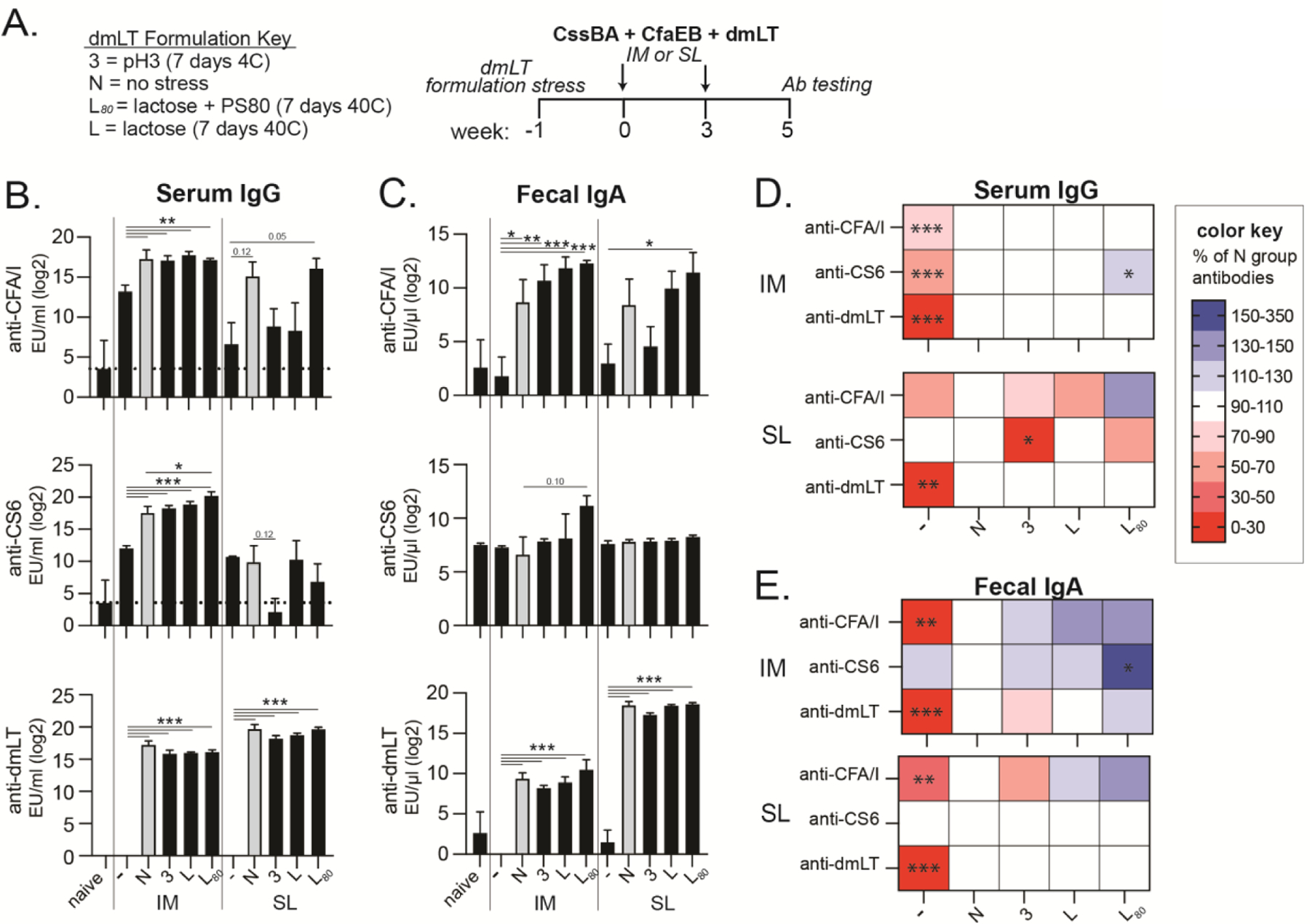
ETEC serum IgG and fecal IgA titers following intramuscular or sublingual administration of CssBA, CfaEB, and dmLT post thermal or chemical stressor. BALB/c mice were either left naïve or immunized twice (week 0 and 3) with 2.5 μg of each CssBA and CfaEB via IM or SL delivery with 0.1 μg dmLT for the IM route and 5 μg dmLT for the SL route. Mice were sacrificed 2 weeks after the last immunization, and serum and feces were collected for antibody analyses. **(A)** Schematic of immunization schedule. **(B)** Serum anti-CFA/I, anti-CS6, and anti-dmLT IgG by EU/ml. **(C)** Fecal anti-CFA/I, anti-CS6, and anti-dmLT IgA by EU/μl. **(D)** Heat maps from IM groups presented as the % change in antibody levels compared to no stress dmLT group. **(E)** Heat maps from SL groups presented as the % change in antibody levels compared to no stress dmLT group. Bar values are represented as mean + SEM with significance indicated as *P ≤ 0.05, **P ≤ 0.01 or ***P ≤ 0.001 by Bonferroni’s multiple comparison (ELISA) or Kruskal-Wallis uncorrected Dunn’s (heatmaps) post-hoc tests.

dmLT formulations were again tested with no stress (N), with pH 3 (3), or with thermal stress (40C for 7 days) in L or L+PS80 buffers. These conditions were chosen to represent destabilizing and stabilizing conditions. These dmLT formulations were evaluated by SDS-PAGE, GM1 binding ELISA and ARGEN light scattering (Fig. 6) at the same time as immunizing animals with antigen (Fig. 7). The pH3 formulation dissociated the AB_5_ molecule to A and B-subunits as expected and by detection of higher levels of B_1_ monomer by SDS-PAGE, by poor dmLT detection in the GM1-binding ELISA, and by reduced molecular weight analysis in ARGEN light scattering. dmLT formulations in L+PS80 with thermal stress preserved detection of both A and B bands, but induced aggregation by light scattering analysis which was not observed to same extent as L alone, similar to Figure 2.

Concurrent to biochemical analyses, we immunized mice with 2.5μg CssBA and 2.5μg CfaEB antigens with or without the addition of the modified dmLT formulations by IM or SL routes. dmLT was used at 0.1 μg for IM or 5 μg for SL vaccination. Mice were boosted at week 3 and sacrificed at week 5 for fecal and serum antibody analyses to CFA/I (the colonization factor of CfaEB), CS6 (the colonization factor of CssBA), and dmLT (Fig. 7A, Supplemental Fig.3). We observed that mice immunized with dmLT (no stress group, grey bars) exhibited higher levels of serum IgG and fecal IgA than antigen only group (Fig.7B–E) as expected based on its immunogenic and adjuvant properties. Serum or fecal anti-dmLT antibody development was robustly observed in all SL or IM dmLT formulations, though small differences in magnitude of antibodies were evident particularly in the pH3 groups. dmLT formulations with pH and thermal stress were able to maintain adjuvant activity, including generation of antibody responses to co-administered CfaEB and CssBA antigens by IM delivery that was similar or better (e.g. L+PS− 80, 7 days, 40C IM group) than no stress dmLT group (Fig. 7B–E). However, both pH and thermal stress L groups resulted in significant loss of dmLT adjuvant activity by SL vaccination, most clearly indicated by reduced anti-CFA/I serum IgG and fecal IgA. We also observed an unexpected detection of highest fecal anti-CFA/I, -CS6, -dmLT IgA responses with stressed dmLT groups, including the L+PS80 with thermal stress with evidence in both IM and SL routes (Fig. 7C,D,E). Minimal serum IgA was detected except against dmLT, but also in L+PS80 with thermal stress exhibited the highest anti-dmLT IgA after IM or SL delivery (Supplemental Figure 3).

Generation of these ETEC-specific antibody responses (Fig. 7) was impacted strongly by both antigen and route, similar to previous reports [24]. For example, serum IgG responses to CFA/I and CS6 tended to be higher or consistently detected after IM delivery than SL delivery; whereas, SL delivery was associated with higher dmLT serum IgA and fecal IgA than IM. For antigen responses, fecal IgA responses were more often detected to CFA/I than to CS6 antigen. In addition, CS6 serum IgG and fecal IgA antibody responses were poorly detected with most SL delivered formulations, whereas CFA/I were clearly detected (in compared to naïve or antigen only groups).

**Figure 8.**
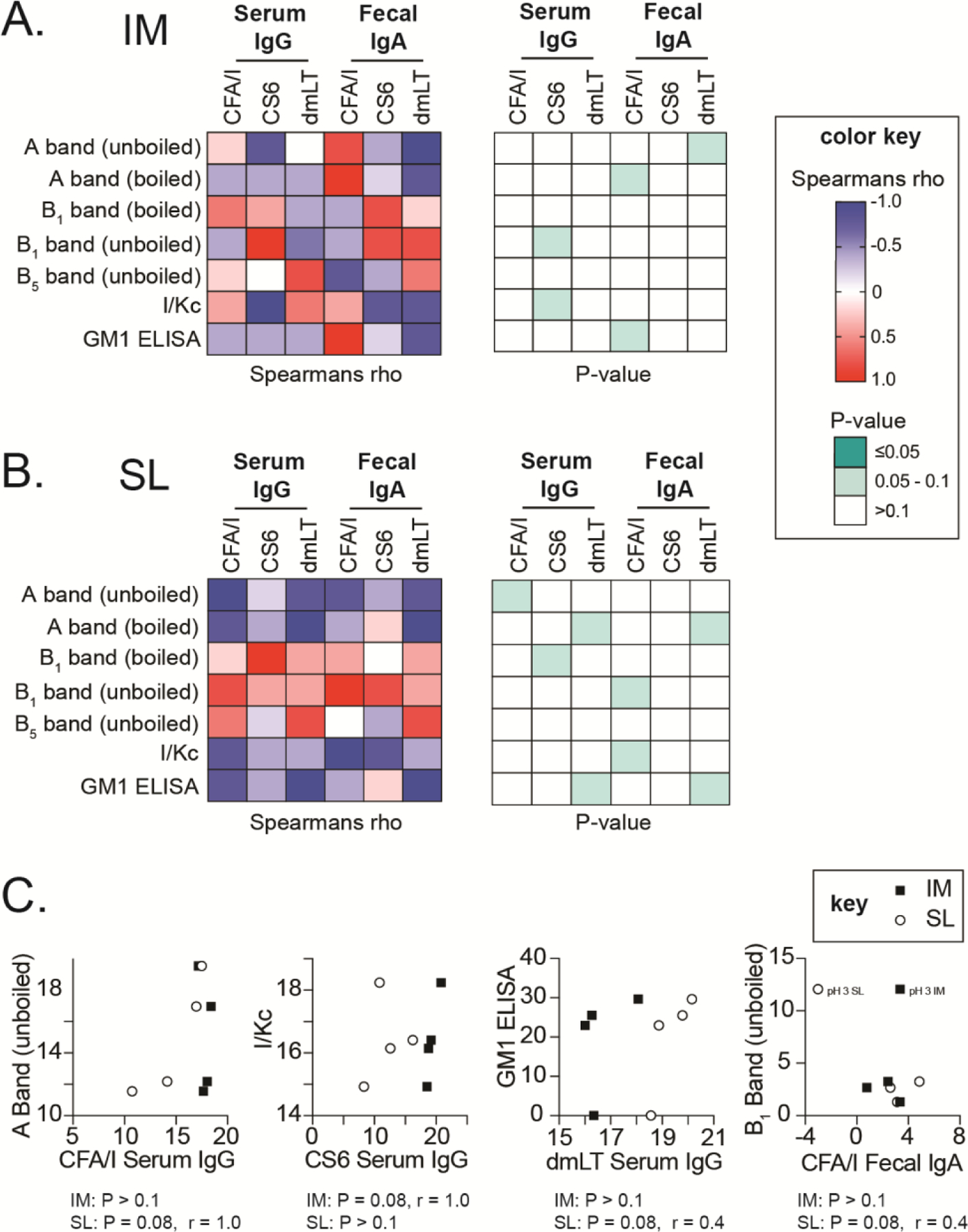
Correlation matrices among immune responses and biochemical analyses of dmLT formulations from the ETEC mouse model. Heatmaps of Spearman’s rank correlation (rho) and p-values for indicated humoral analysis post-ETEC vaccination and dmLT biochemical analyses. (A) Intramuscularly immunized mouse antibody responses heatmaps. (B) Sublingually immunized mouse antibody responses heatmaps. (C) Select graphs of individual correlations with each immunization/formulation group represented by square (IM groups) or open circle (SL groups).

To investigate the possible drivers of immune responses as it relates to alterations in dmLT formulations, we analyzed correlations between antibody levels and biochemical changes to dmLT in these formulations (Fig.8A–C). No one biochemical test predicted immune responses to vaccination reliably by route or antigen. Noticeably, there was a clear route and antigen effect. Select serum IgG or fecal IgA responses were correlated to biochemical measures (P = 0.083) as indicated. As with polio studies, GM1 ELISA was positively correlated related to anti-dmLT IgG, but only for SL and not for IM. A band also positively correlated with development of antigen-specific IgG or IgA but both routes, but not consistently for antigens or even for gel running conditions (e.g. boiled vs unboiled samples). Similar findings were observed for B band and I/Kc measures of molecular weight. Taken together these results confirm that biochemical alterations to dmLT by formulation or stress have limited impacts on vaccine efficacy. We conclude from both polio and ETEC studies that route as well as immunization antigen are extremely important in vaccine outcomes with dmLT, even over biochemical stress applied directly to dmLT (e.g. pH, heat).

## DISCUSSION

A major goal of vaccine development is to minimize composition changes (e.g., aggregation, dissociation, etc.) while optimizing beneficial impacts on immunogenicity. These changes should be well understood and, if beneficial, controlled for reproducibility. While the addition of biochemical excipients has been investigated to stabilize vaccines adjuvants, rarely are these evaluations linked to vaccination outcomes relying on potency assays and biochemical assays. Here, we followed up on recent reports on dmLT toxoid and adjuvant for buffer composition and thermal stability [38, 39]. We investigated stabilizing and destabilizing formulations with or without thermal stress for dmLT immunogenic and adjuvant properties by linking three biochemical assays to vaccine outcomes in ETEC and polio models. These results have implications for LT-based vaccines and general experimental approaches for optimizing vaccine stability.

Our major finding was that biochemical assessment of dmLT reliably predicted vaccine efficacy either in dmLT toxoid immunogenicity or adjuvant effects. We began by investigating the biochemical analyses of dmLT with combined with buffers that included sugar excipients lactose or sucrose, the surfactant PS80 or a lowered pH (Figure 2). We subjected select formulations to thermal stress for up to 7 days. Formulations with thermal stress or pH3 had evidence of destabilized protein structure, by A-subunit band analysis, GM1-binding ELISA (which requires the intact AB_5_ configuration), and light scattering assessment of aggregation or dissociation (Fig.2, Fig.6). Lactose with the addition of the PS80 surfactant exhibited the most protection from thermal-induced changes but also exhibited greatest aggregation of the protein. However, when these formulations were tested in vaccination models, correlations from these dmLT formulation biochemical analyses were not consistently significantly to the development of antibody responses to dmLT or co-administered antigen post-vaccination (Fig.5, Fig.8).

Vaccination with dmLT induced both anti-dmLT or -antigen IgG or IgA using either IPOL, CfaEB, or CssBA antigens. Our second major finding was that vaccine delivery route was more important to post-vaccination antibody responses than dmLT biochemical stability, similar to past mouse studies [23, 24]. Parenteral vaccination was far less sensitive than mucosal vaccination to biochemically destabilizing formulations or stress (e.g. serum CFA/I IgG responses shown in Fig.7B–E). The A1 domain of dmLT and its parent molecule LT has adjuvant activity by itself that is enhanced with free B-subunit [6, 13, 49, 50], indicating the AB_5_ conformation is likely not critical for maintain parenteral vaccine responses. However, at mucosal surface the B-subunit promotes mucosal binding and entry [3, 51], which is likely a key factor in how sensitive the protein is to biochemical changes and mucosal vaccination outcomes. The mechanisms of action are likely different between dmLT adjuvant vaccine delivery by parenteral injection in the skin, muscle, or to the mucosa under the tongue. For example, the skin is known to have more dendritic cell populations than muscle and dmLT injection actively stimulates their recruitment to the site of injection [20], and specific skin antigen presenting cells have been critical for intradermal vaccine responses [52]. Other studies have shown epithelial cells and fibroblasts in specific mucosal compartments can play critical roles in immune signaling and generation of adaptive immunity with adjuvants [49, 53]. Understanding which cellular targets and innate signaling pathways are activated at these routes will be important to undertake for a clearer understanding on how dmLT, route, and antigen combinations shape immunity.

In these studies, IPOL and ETEC vaccine antigens were uniquely impacted by formulation and biochemical stressors. Meaning, we observed loss of responses to PV serotype 2 neutralization that were not concurrent to changes with PV serotype 1 or 3 (Fig.4). Similarly, A-band detection by SDS-PAGE analysis of formulations was correlated to PV serotype 3 neutralizing antibodies but not to other serotypes (Fig.5). CFA/I and CS6 antibody responses to ETEC antigens CfaEB and CssBA, respectively, also seemed to be distinctly induced. CS6 antibody responses were only robustly observed after IM and not SL immunization, whereas CFA/I responses were observed with both IM and SL. However, both antigens were given at the same doses for these routes. Additional changes in dosing of antigen (or adjuvant) likely could impact the CS6 antibody responses to SL vaccination.

Taken together, we cannot recommend any one formulation for use in dmLT vaccines to enhance stability and vaccine responses. However, specific formulation changes in dmLT may have implications for biasing immunity after vaccination. Aggregated formulations with L+PS80 having undergone thermal stress for 7 days consistently induced more fecal IgA in the ETEC mouse model after both IM and SL routes (Fig.7). An area of interest in potential future studies with dmLT might evaluate the combination of parenteral and mucosal vaccination routes with stabilized or destabilized formulations to significantly enhance mucosal immune responses which are thought to be critical for enteric pathogens. ETEC accounts for 14,000–42,000 deaths annually in children under 5 years of age [54]. Both travelers and deployed US military personnel are also vulnerable populations to ETEC-induced diarrhea and potential disease complications [55, 56]. Furthermore, diarrheal illness contributes to malnutrition, stunted growth, impaired cognitive development, and high morbidity rates, affecting 20% of children world-wide [57]. Thus, if aggregated dmLT helps to achieve a stronger fecal antibody response, then likely protection against this intestinal pathogen would be enhanced or be more durable.

A potential limitation of this study is that though stabilizing and destabilizing formulations were evaluated, an extensive evaluation of buffer excipients, compositions, stressors, or storage times and temperatures was not conducted. We focused our evaluations on biochemical assays currently used for the GMP drug product analysis or bedside mixing formulation analyses used in ongoing clinical trials (e.g. SDS-PAGE, GM1-binding ELISA) or classic biochemical analyses (e.g. light scattering). Future studies should focus on immunological evaluations (e.g. antigen presenting cell activation or cytokine release) similar to Suppl. Fig.1C that may provide better surrogates of *in vivo* efficacy and stability without requiring animal studies and better correlated with human PBMC or dendritic cell studies [13, 37, 58]. In addition, formulations were admixed with antigen in PBS before immunization rather than being injected directly into mice, which would alter final pH, sugar or PS80 concentration in these formulations. Promising thermal stability was observed with Lactose with PS80 (Figure 4, 7), as well as minimal skin reactogenicity (Fig.3), though curiously effects on vaccine-induced antibody responses were better after 7 days of thermal stress than 3 days. Thus, given these considerations, we would recommend that additional evaluations be performed for advance candidate vaccines using dmLT, rather than extrapolation of past formulation analyses. In conclusion, we show that optimal conditions for dmLT formulation are route and antigen specific, rather than severely impacted by biochemical stability of the protein. Furthermore, taken together these results indicate that dmLT is a robust molecule, particularly when used for parenteral vaccination.

## Supplementary Material

Supplementary material

## Figures and Tables

**Table 1.** dmLT Formulation for Polio and ETEC studies.^1^

Polio Formulations				
Name	pH	Salts, amino acids	Sugars	Polysorbate-80
PBS	7.4	137 mM NaCl, 2.7 mM KCl, 8.1 mM NaPO_4_, 1.5 mM KPO_4_	none	0.05%
Low pH	3–4	139.5 mM NaCl, 2.2 mM KCl, 10.5 mM NaPO_4_, 1.2 mM KPO_4_ HCL (to pH3–4)	none	0.05%
L	7.2–7.4	83 mM NaCl 44.5 mM NaPO_4_, 10.7 mM KPO_4_	5% lactose	none
L_80_	7.2–7.4	82 mM NaCl, 44.5mM NaPO_4_, 10.7mM KPO_4_	5% lactose	0.05%
S_80_	7.2–7.4	50 mM NaCl 50 mM NaPO_4_, 10.7 mM KPO_4_ 5mM methionine	10% sucrose	0.1%
ETEC formulations				
Name	pH	Salts, amino acids	Sugars	Polysorbate-80
PBS	7.4	137 mM NaCl, 2.7 mM KCl 11.9 mM phosphates (Fisher BP6651)	none	none
Low pH	3–4	137 mM NaCl, 2.7 mM KCl, 11.9 mM phosphates (Fisher BP6651) HCl (to pH3)	none	none
L	7.2–7.4	83 mM NaCl 44.5 mM NaPO_4_, 10.7 mM KPO_4_	5% lactose	none
L_80_	7.2–7.4	82 mM NaCl, 44.5mM NaPO_4_, 10.7mM KPO_4_	5% lactose	0.05%

1 Polio formulations were made and frozen prior to thawing and use; ETEC formulations were prepared and freshly evaluated, matching between all biochemical and immunization studies.

## Data Availability

Data from this study is accessible upon reasonable request to the authors.

## HIGHLIGHTS

- Formulations and thermal stress uniquely alter dmLT biochemical stability
- Biochemical stability poorly predicts immunity in mouse dmLT vaccine models
- Delivery route and antigen impact vaccine outcomes more than dmLT stability
- Biochemical and immunity testing are needed for vaccine formulation optimization
