# Supplementary material for: Route and antigen shape immunity to dmLT-adjuvanted vaccines to a greater extent than biochemical stress or formulation excipients
Source: Vaccine. Author manuscript; Available in PMC 2023 Jun 29. (PMC10308557; doi:10.1016/j.vaccine.2023.01.033)
Supplement: Supplementary material [file NIHMS1910132-supplement-Supplementary_material.pdf]

Supplemental Figures and Figure Legends

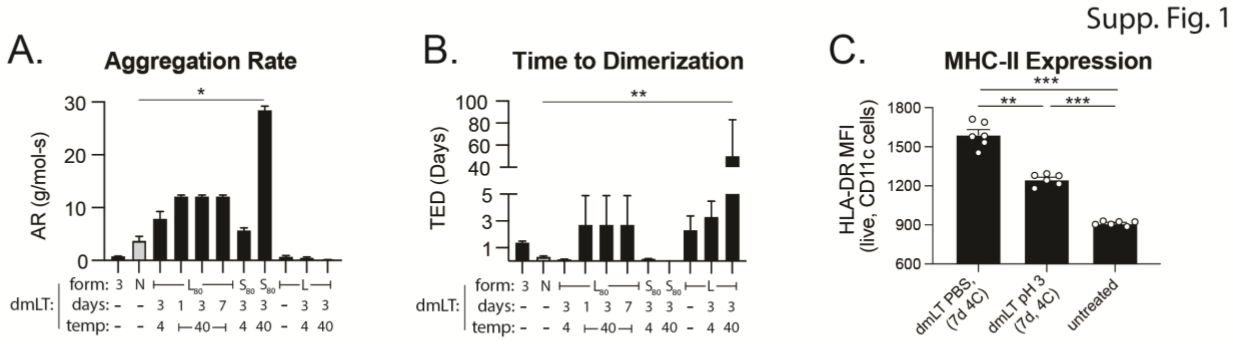

Supplemental Figure 1. Additional Light Scattering data for dmLT formulations used in Polio vaccination experiments. **(A)** Aggregation rate of formulations calculated by taking the derivative of I/Kc values, shown in seconds^{-1}. (B) Time to dimerization for each formulation, shown in days. (C) Mean Fluorescent intensity of HLA-DR expression in live, CD11c+ gated THP-1 cells treated for 24h with indicated treatment (following previously reported methods [7]). Values are represented as mean + SEM with significance indicated as *$P \leq 0.05$, **$P \leq 0.01$ or ***$P \leq 0.001$ by ANOVA using using Bonferroni's multiple comparison (HLA-DR MFI) or Kruskal-Wallis uncorrected Dunn's (AR, TED) post-hoc test.

Supp. Fig. 2

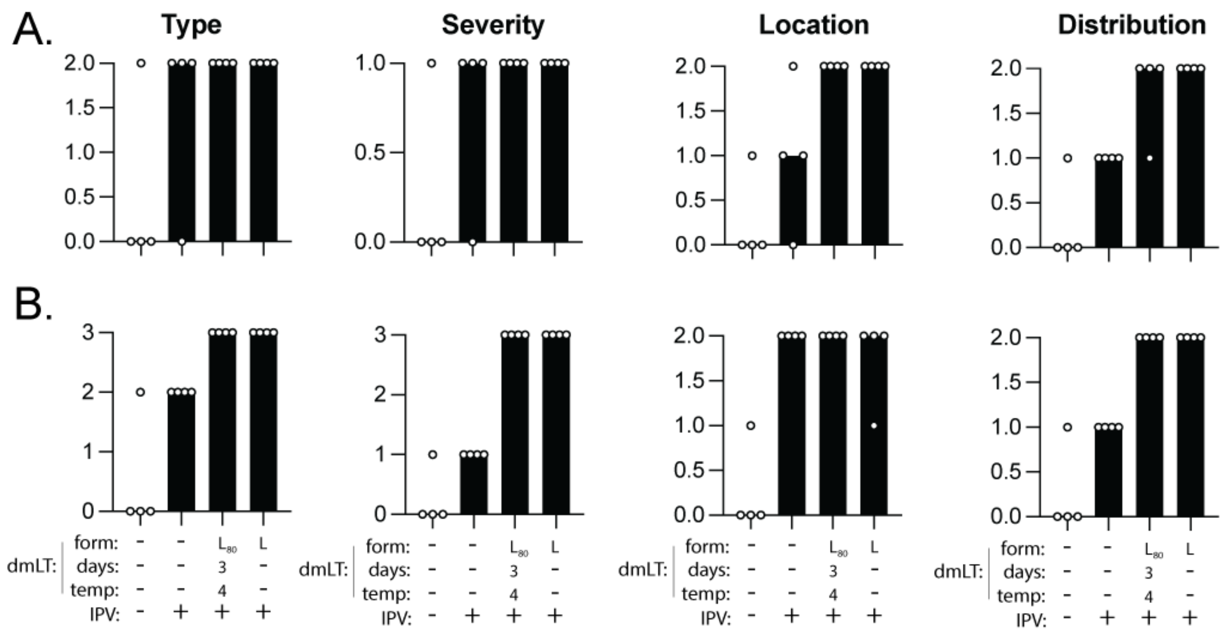

Supplemental Figure 2. Additional skin scoring analysis. Individual histological scores for prime (A) and booster (B) skin tissue sections used to make the histology composite score including: type (1=acute/neutrophilic infiltration, 2=chronic/lymphoplasmacystic infiltration, 3=both), severity (1=minimal/mild, 2=moderate, 3=severe), location (1=dermis, 2=subcutis, 3=both), and distribution of inflammation (1=localized, 2=diffuse).

Supp. Fig. 3

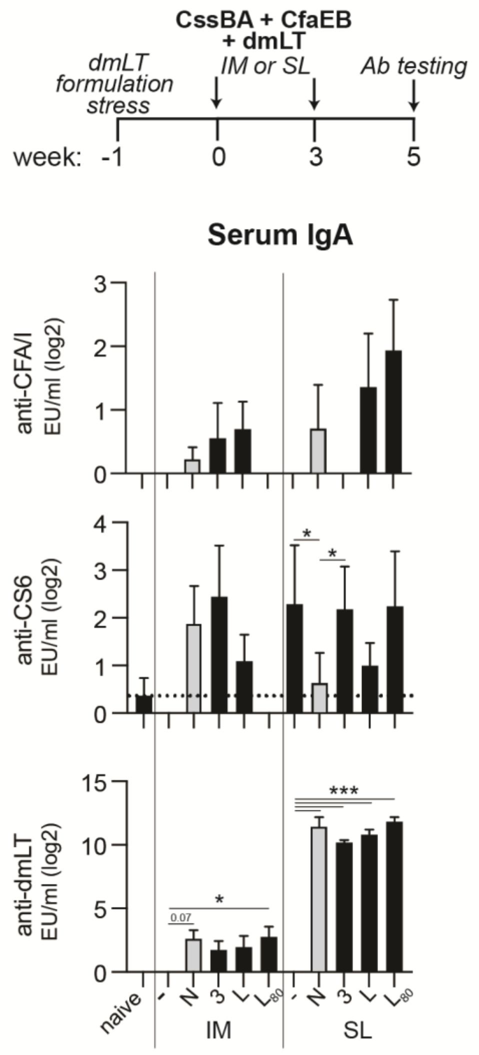

Supplemental Figure 3. ETEC serum IgA titers following intramuscular or sublingual administration of CssBA, CfaEB, and dmLT post-dialysis in various buffers. BALB/c mice were either left naïve or immunized twice (week 0 and 3) with 2.5 µg of each CssBA and CfaEB via IM or SL delivery with 0.1 µg dmLT for the IM route and 5 µg dmLT for the SL route. Mice were sacrificed 2 weeks after the last immunization, and serum were collected for antibody analyses. Serum anti-CFA/I, anti-CS6, and anti-dmLT IgA by EU/ml. Bar values are represented as mean + SEM with significance indicated as **P* ≤ 0.05, ***P* ≤ 0.01 or ****P* ≤ 0.001 by Bonferroni's multiple comparison post-hoc tests.
